# Job satisfaction, life satisfaction, and associated factors among hospital nurses: a cross-sectional study in Türkiye

**DOI:** 10.1038/s41598-025-85564-4

**Published:** 2025-02-17

**Authors:** Volkan Medeni, İrem Medeni, Gizem Altunay, Asiye Uğraş Dikmen, Mustafa Necmi İlhan

**Affiliations:** 1 Department of Public Health, Faculty of Medicine, Gazi University, Ankara, Türkiye Turkey; 2 Employee Health Department, General Directorate of Public Health, Ministry of Health, Ankara, Türkiye Turkey

**Keywords:** Job satisfaction, Life satisfaction, Nurses, Mental health, Healthcare, Working conditions, Health services, Occupational health, Public health, Health occupations

## Abstract

Job satisfaction strongly affects nurses’ life satisfaction and is directly affected by life satisfaction. Our study aimed to determine nurses’ life and job satisfaction, show their relationship, and evaluate the factors affecting them. This cross-sectional study was conducted in 2022 at a university hospital in Türkiye. The study population included all nurses working at the hospital for at least one month, and 920 nurses participated. Data were collected using a structured questionnaire, which consisted of sections on sociodemographic characteristics, job and life satisfaction, and factors related to the nursing profession. Job satisfaction was measured using the Index of Job Satisfaction, while life satisfaction was assessed with the Life Satisfaction Scale, both validated tools. Data collection occurred during periodic health examinations through face-to-face interviews. Most participants chose the nursing profession willingly and found it suitable for themselves. However, many reported dissatisfaction with their earnings. Higher job satisfaction was associated with older age, having children, good perceived health, shorter weekly working hours, willingly choosing the nursing profession and unit, and favorable working conditions and income. Similarly, life satisfaction was higher among those with good perceived health, fewer weekly working hours, willingly chosen profession and unit, and no smoking or chronic diseases. Supportive working conditions and adequate income strongly influenced both job and life satisfaction. A significant, positive, and moderate correlation was found between job and life satisfaction, highlighting their interconnectedness. These findings suggest that improving nurses’ working conditions, ensuring adequate income, and supporting healthier lifestyles could enhance job and life satisfaction. Enhancing working conditions is essential to improving nurses’ job satisfaction, which, in turn, positively impacts their overall life satisfaction. Policymakers should prioritize initiatives that address workplace challenges and foster a supportive environment for nurses.

## Introduction

Job satisfaction is a multifaceted structure that includes feelings such as satisfaction or dissatisfaction arising from employees’ work experiences, emotional and positive attitudes toward one’s job, and perceived love^1^. It consists of various components, including work conditions, wages, promotion opportunities, quality of work, organizational policies, appreciation, security, and interpersonal communication^2^. Job satisfaction affects both working life and every aspect of human life^3^. Satisfaction with one’s job significantly affects both life satisfaction and productivity^4^. Professional activities constitute an essential part of life for most people and contribute to general life satisfaction^5^. Life satisfaction, a difficult concept to define due to its ambiguous, multidimensional, and complex structure, is the state of balance between the current situation and personal expectations^6,7^. The more our needs are met, the more satisfied we are with life^8^. Many factors, such as social life, cultural activities, family, psychological characteristics, expectations, and beliefs, affect life satisfaction^9^. Life satisfaction level is closely related to job satisfaction, which is affected by both the working environment and individual factors^10^.

Job satisfaction is essential for nurses, who are indispensable in protecting and improving human health, providing quality health care, and reducing turnover^11^. Low job satisfaction among healthcare workers may affect the quality of patient care and employee performance, leading to negative results such as absenteeism, frequent turnover, and low performance^12^. Nurses often have to work under conditions such as patient-related problems, heavy and intensive workloads, staff shortages, aging nurse workforce, ineffective policies supporting nurses in administrative systems, unfair wages, poor working conditions, lack of resources and equipment, limited career and training opportunities^13^. When nurses are analyzed in terms of burnout, job stress, job satisfaction, and life satisfaction, it is seen that their positive thinking capacities and job satisfaction decrease, and they experience physical, mental, and social problems because of the problems arising from their profession^14^. Job satisfaction strongly affects nurses’ life satisfaction and well-being and is directly affected by life satisfaction^15^.

The relationship between nurses’ job and life satisfaction impacts their mental health, motivation, and performance. Our study aimed to determine nurses’ life and job satisfaction levels and show their relationship. We also intend to evaluate the factors affecting these concepts. Our results may contribute to developing policies and practices to improve nurses’ working conditions and quality of life.

## Materials and methods

The study is cross-sectional type. It was conducted in 2022 at Gazi University Faculty of Medicine Hospital, one of the largest university hospitals in Türkiye. This hospital is an inpatient treatment institution with emergency services, polyclinics, additional service buildings, laboratories, and imaging units. It is an important education, research, and application center where expert teams perform advanced diagnosis and treatment of many diseases in internal and surgical branches. The study population was all the nurses in the university hospital for at least one month. No sample selection was made, aiming to reach all 947 people constituting the population. All nurses who provided informed consent were included in the study. Nine hundred twenty nurses participated in the study. The access percentage was 97.1%.

The data collection form of the study consists of four sections and includes 28 questions in total. In the first part, there are questions about sociodemographic characteristics; in the second part, there are questions about nursing. The third section includes the Index of Job Satisfaction, and the fourth includes the Life Satisfaction Scale.

Our research variables obtained from the personal files of the participants were gender, age, marital status, number of children, education level, smoking, chronic disease presence, total and weekly working time, and working department. According to the participants’ statements, information was obtained about perceived health status, willingly choosing the working department or the nursing profession, finding the nursing job suitable for oneself, and thinking that monthly earnings are sufficient. Two scales were applied to assess job and life satisfaction.

The Index of Job Satisfaction was developed by Brayfield and Rothe in 1951. It is a Likert-type self-assessment scale. The original form contains 18 items. In 1998, Judge et al. created a 5-item short form of the scale. This form has been used more frequently over time. Each item is scored on a five-point scale (1–5; strongly disagree-strongly agree). The short form of the scale, adapted into Turkish by Keser and Bilir in 2019, is a valid and reliable measurement tool for measuring employees’ satisfaction with their jobs. The Life Satisfaction Scale was developed by Diener et al. in 1985. It is a valid and reliable scale used to analyze life satisfaction. It is unidimensional and consists of 5 items. The subjects were asked to rate each item from 1 to 7 on the scale. When the seven graded options were converted into points, they were rated as inappropriate 1, not appropriate 2, somewhat not appropriate 3, neither appropriate nor not appropriate 4, somewhat appropriate 5, appropriate 6, and very appropriate 7. The scale was adapted into Turkish by Köker in 1991 and used by different researchers.

In determining the perceived health status, the question “How is your health status in general?” was asked, evaluation was made with a five-point Likert scale, and the categories of “moderate,” “poor,” and “very bad” were defined as the health status perceived negatively.

The study was performed in accordance with the Declaration of Helsinki. It was ethically approved by the Institutional Review Board of the Gazi University Ethical Committee (Approval no. 2022 − 587). Informed consent was obtained. There was no conflict of interest within the scope of the study. The questionnaire forms were applied to the nurses working in the hospital by face-to-face interview method during periodic health examinations by the researchers. The average time to answer a questionnaire was 5 min.

Statistical analyses in the study were performed using the Statistical Package for Social Science for Windows 23.0 program. Under the heading of descriptive statistics, categorical variables were expressed with numbers and percentages; numerical variables were expressed with mean, standard deviation, and median (maximum-minimum). Categorical variables were compared using the Pearson chi-square test, Fisher’s exact test, and Yates continuity correction. Compliance with normal distribution was checked using Kolmogorov-Simirnov and Shapiro-Wilk tests. It was observed that parametric assumptions still needed to be met. Mann-Whitney U test was used to test the significance of the difference between the means obtained from two independent sample groups. Spearman’s rank correlation was performed to determine the degree and direction of the linear relationship between the variables. The statistical significance level was accepted as *p* < .05.

## Results

A total of 920 nurses participated in the study, with an average age of 36.32 ± 8.92 years (range: 23–67). On average, participants had worked at the hospital for 13.49 ± 9.59 years (range: 1 month–42 years) and in their current unit for 5.76 ± 6.30 years (range: 1 month–34 years). Of the 27 nurses excluded from the study, 15 declined participation due to unwillingness to undergo periodic health checks, seven were excluded due to shift work, three were on leave, and two refused to participate.

Most participants were women (92.2%) and university graduates (85.1%). Most were married (60.0%), with 45.8% having no children. A significant proportion (56.0%) had never smoked, and 27.7% had chronic diseases. Regarding health status, 57.9% described it as good, while 26.1% considered it moderate (Table 1).

**Table 1:** Demographic characteristics of participants.

	Number (*n*)	Percentage (%)
Gender (*n* = 920)		
Women	848	92.2
Men	72	7.8
Age (*n* = 920)		
Less than 30 years old	314	34.1
30–39 years old	254	27.6
40–49 years old	291	31.7
50 years old and over	61	6.6
Marital status (*n* = 920)		
Married	552	60.0
Single	318	34.6
Divorced/Widowed	50	5.4
Number of children (*n* = 920)		
0	421	45.8
1	196	21.3
2	267	29.0
3 and above	36	3.9
Graduation (*n* = 920)		
High school	12	1.3
Junior college	41	4.5
College	783	85.1
Master’s/PhD	84	9.1
Smoking (*n* = 920)		
Never smoked	515	56.0
Quit smoking	122	13.2
Smoking	283	30.8
Chronic disease (*n* = 920)		
There is	255	27.8
None	665	71.2
Perceived health status (*n* = 920)		
Very good	129	14.0
Good	533	57.9
Average	241	26.1
Bad	15	1.6
Very bad	2	0.2

Participants worked in various departments: 24.5% in internal wards, 21.1% in intensive care, 17.1% in surgical wards, and 12.8% in outpatient clinics. 64.3% worked 40 h or less per week. Regarding tenure, 32.4% had been at the hospital for 5 years or less, and 26.5% for over 20 years. In their current unit, 53.6% chose their department willingly. Most participants (75.2%) willingly chose nursing as a profession, and 78.4% found it suitable. 88.0% felt their earnings needed to be increased. 57.9% worked day and night shifts, and 33.5% described their working conditions as good (Table 2).

**Table 2:** Characteristics of the participants’ jobs.

	Number (*n*)	Percentage (%)
Department (*n* = 920)		
Internal ward	225	24.5
Intensive care	194	21.1
Surgical ward	158	17.1
Outpatient clinic	118	12.8
Operating room	75	8.1
Emergency service	51	5.6
Administrative unit	29	3.2
Imaging	28	3.0
Dialysis/Chemotherapy unit	21	2.5
Other*	19	2.1
Duration of employment in the hospital (*n* = 920)		
Less than 1 year	42	4.6
1–5 years	256	27.8
6–10 years	101	11.0
11–15 years	137	14.9
16–20 years	140	15.2
More than 20 years	244	26.5
Weekly working hours (*n* = 920)		
40 h and less	592	64.3
More than 40 h	328	35.7
Duration of employment in the department (*n* = 920)		
Less than 1 year	150	16.3
1–2 years	230	25.0
3–4 years	156	17.0
5–9 years	179	19.4
10 years and more	205	22.3
Willingly choosing the department (*n* = 920)		
Yes	493	53.6
No	427	46.4
Shift (*n* = 920)		
Day shift only	351	38.2
Day shift + night shift	533	57.9
Night shift only	36	3.9
Working conditions (*n* = 920)		
Very good	31	3.4
Good	308	33.5
Average	407	44.2
Bad	146	15.9
Very bad	28	3.0
Willingly choosing the profession (*n* = 920)		
Yes	692	75.2
No	228	24.8
The profession is suitable for her/him (*n* = 920)		
Yes	721	78.4
No	199	21.6
Monthly earnings are sufficient (*n* = 920)		
Yes	110	12.0
No	810	88.0

* Other: Stem cell transplantation unit. bone marrow transplantation unit. infection control committee. therapeutic apheresis centre. blood centre. haemovigilance. central nutrition unit.

Table 3 shows that job satisfaction scores were significantly higher among nurses aged 40 or older, those with children, those with good perceived health, those who did not work night shifts, and those who worked fewer than 40 h per week. Additionally, nurses who had worked at the hospital for more than 10 years willingly chose the nursing profession and found the profession suitable for themselves reported higher job satisfaction. Nurses who worked outside the ward had been in their current unit for five or more years and willingly chose their unit also had higher scores. Furthermore, those who evaluated their working conditions as good and those who found their income sufficient had notably higher job satisfaction scores.

**Table 3:** The job satisfaction scale scores according to some characteristics of participants.

		The job satisfaction scale				
		M	SD	MR	U	p*
Gender (*n* = 920)	Women (*n* = 848)	17.15	4.26	459.44	29625.50	0.676
	Men (*n* = 72)	17.42	3.87	473.03		
Age (*n* = 920)	40 years old and above (*n* = 352)	17.77	4.16	502.90	85042.00	< 0.001*
	Less than 40 years old (*n* = 568)	16.80	4.23	432.22		
Marital status (*n* = 920)	Married (*n* = 552)	17.24	4.19	465.47	98824.50	0.486
	Not married (*n* = 368)	17.07	4.30	453.04		
Having children (*n* = 920)	Yes (*n* = 499)	17.43	4.12	476.62	96998.00	0.045*
	No (*n* = 421)	16.87	4.35	441.40		
Smoking (*n* = 920)	No (*n* = 637)	17.11	4.37	458.39	88794.00	0.718
	Yes (*n* = 283)	17.31	3.91	465.24		
Chronic disease (*n* = 920)	No (*n* = 665)	17.17	4.22	460.30	84652.00	0.970
	Yes (*n* = 255)	17.17	4.26	461.03		
Perceived health status (*n* = 920)	Very good/good (*n* = 662)	17.55	4.20	483.38	70250.00	< 0.001*
	Average/bad/very bad (*n* = 258)	16.21	4.18	401.79		
Night shift (*n* = 920)	No (*n* = 351)	17.78	4.28	503.14	84894.00	< 0.001*
	Yes (*n* = 569)	16.80	4.16	434.20		
Weekly working hours (*n* = 920)	40 h and less (*n* = 592)	17.69	4.22	495.06	76630.50	< 0.001*
	More than 40 h (*n* = 328)	16.24	4.11	398.13		
Duration of employment in the hospital (*n* = 920)	More than 10 years (*n* = 521)	17.68	4.06	494.31	86322.00	< 0.001*
	10 years and less (*n* = 399)	16.51	4.36	416.35		
Willingly choosing the profession (*n* = 920)	Yes (*n* = 692)	17.73	4.03	494.64	55263.50	< 0.001*
	No (*n* = 228)	15.48	4.38	356.88		
The profession is suitable for her/him (*n* = 920)	Yes (*n* = 721)	18.14	3.74	519.41	29266.00	< 0.001*
	No (*n* = 199)	13.68	4.07	247.07		
Department (*n* = 920)	Not clinics (*n* = 486)	17.46	4.21	478.05	96931.00	0.033*
	Clinics (*n* = 434)	16.85	4.23	440.84		
Duration of employment in the department (*n* = 920)	5 years and above (*n* = 384)	17.52	4.11	483.48	94087.00	0.026*
	Less than 5 years (*n* = 536)	16.92	4.30	444.04		
Willingly choosing the department (*n* = 920)	Yes (*n* = 493)	18.11	4.13	523.18	74353.50	< 0.001*
	No (*n* = 427)	16.09	4.09	388.13		
Working conditions (*n* = 513)	Very good/good (*n* = 339)	19.46	3.53	462.52	38807.50	< 0.001*
	Bad/very bad (*n* = 174)	13.68	4.29	299.35		
Monthly earnings are sufficient (*n* = 920)	Yes (*n* = 110)	19.38	3.54	605.91	28554.50	< 0.001*
	No (*n* = 810)	16.88	4.23	440.75		

M: Mean; SD: Standard deviation; MR: Mean rank; U: Mann-Whitney U value.

*p: Statistically significant.

Table 4 shows that life satisfaction was higher among nonsmokers, those without chronic diseases, and those with good perceived health. Nurses who worked 40 h or less per week, who willingly chose the nursing profession, and who found the profession to be a good fit for them reported higher levels of life satisfaction. Those who willingly chose their work unit and evaluated their working conditions as good also had higher life satisfaction scores. Additionally, those who found their income sufficient had significantly higher life satisfaction than those who found their income insufficient.

According to the scale scores, a significant, positive, and moderate relationship exists between job and life satisfaction (*r* = .51; *p* < .001).

**Table 4:** The satisfaction with life scale scores according to some characteristics of participants.

		The satisfaction with life scale				
		M	SD	MR	U	p*
Gender (*n* = 920)	Women (*n* = 848)	19.01	6.28	458.21	28583.00	0.382
	Men (*n* = 72)	19.86	6.39	487.51		
Age (*n* = 920)	40 years old and above (*n* = 352)	19.17	6.31	466.61	97816.00	0.582
	Less than 40 years old (*n* = 568)	19.02	6.29	456.71		
Marital status (*n* = 920)	Married (*n* = 552)	19.26	6.31	468.69	97046.00	0.252
	Not married (*n* = 368)	18.80	6.27	448.21		
Having children (*n* = 920)	Yes (*n* = 499)	19.22	6.36	467.08	101756.00	0.413
	No (*n* = 421)	18.91	6.21	452.70		
Smoking (*n* = 920)	No (*n* = 637)	19.38	6.35	476.64	81765.00	0.024*
	Yes (*n* = 283)	18.39	6.11	430.92		
Chronic disease (*n* = 920)	No (*n* = 665)	19.44	6.20	476.49	74151.00	0.003*
	Yes (*n* = 255)	18.11	6.44	418.79		
Perceived health status (*n* = 920)	Very good/good (*n* = 662)	19.87	6.24	494.02	63206.50	< 0.001*
	Average/bad/very bad (*n* = 258)	17.02	5.97	374.49		
Night shift (*n* = 920)	No (*n* = 351)	19.47	6.56	475.36	94642.00	0.182
	Yes (*n* = 569)	18.83	6.11	451.33		
Weekly working hours (*n* = 920)	40 h and less (*n* = 592)	19.48	6.25	478.02	86715.00	0.007*
	More than 40 h (*n* = 328)	18.34	6.31	428.88		
Duration of employment in the hospital (*n* = 920)	More than 10 years (*n* = 521)	19.08	6.34	461.76	103280.50	0.869
	10 years and less (*n* = 399)	19.07	6.24	458.85		
Willingly choosing the profession (*n* = 920)	Yes (*n* = 692)	19.79	6.14	491.13	57692.50	< 0.001*
	No (*n* = 228)	16.89	6.26	367.57		
The profession is suitable for her/him (*n* = 920)	Yes (*n* = 721)	19.96	6.17	497.78	44858.50	< 0.001*
	No (*n* = 199)	15.88	5.67	325.42		
Department (*n* = 920)	Not wards (*n* = 486)	19.01	6.26	458.71	104590.00	0.828
	Wards (*n* = 434)	19.15	6.33	462.51		
Duration of employment in the department (*n* = 920)	5 years and above (*n* = 384)	19.13	6.31	463.04	101937.00	0.806
	Less than 5 years (*n* = 536)	19.04	6.29	458.68		
Willingly choosing the department (*n* = 920)	Yes (*n* = 493)	19.81	6.34	491.89	89781.00	< 0.001*
	No (*n* = 427)	18.23	6.13	424.26		
Working conditions (*n* = 513)	Very good/good (*n* = 339)	21.45	6.15	432.45	49002.50	< 0.001*
	Bad/very bad (*n* = 174)	16.07	6.23	324.40		
Monthly earnings are sufficient (*n* = 920)	Yes (*n* = 110)	22.60	5.19	615.09	27545.00	< 0.001*
	No (*n* = 810)	18.60	6.28	439.51		

*M: Mean; SD: Standard deviation; MR: Mean rank; U: Mann-Whitney U value.

*p::Statistically significant.

## Discussion

Our study aimed to evaluate the job and life satisfaction levels of 920 nurses by investigating various sociodemographic and work-related factors. Our findings showed that factors such as being over 40 years of age, having children, positive perceived health status, not working on night shifts, shorter weekly working hours, working longer as a nurse, willingness to choose the profession and performing department, positive working environment and contentment with income were associated with higher levels of job and life satisfaction among nurses. In addition, a significant positive correlation was observed between job and life satisfaction.

In our study, approximately one-third of the participants were smokers. The Life Satisfaction Scale scores of nonsmokers were found to be higher. In another study conducted in Türkiye, half of the nurses were smokers^16^. According to the Turkish Health Statistics Yearbook, 28% of individuals aged 15 years and over use tobacco and tobacco products every day, and this frequency is lower among women^17^. In a study conducted with women nurses in Iceland, only 8% of the participants said they smoked cigarettes daily^18^. 92% of the nurses participating in a study conducted in England stated that they did not smoke^19^. Smoking may harm life satisfaction by causing health problems. The differences between the studies regarding smoking prevalence may reflect the effectiveness of tobacco control policies between countries and the change in time.

The majority of the participants in our study rated their general health status as good, and approximately one-fourth rated it as moderate. In another study conducted in Türkiye, approximately one-third of the participants stated that their health status was good, and approximately half stated that it was moderate^20^. In a study conducted in Iran, approximately one-third of the nurses rated their health status as good and approximately one-fourth as moderate^21^. Approximately half of the nurses in a study conducted in Israel rated their health status as good, and more than one-third rated it as very good or excellent^22^. Since each country’s health system, lifestyle habits, and socio-economic characteristics are unique, different results are expected to emerge in such evaluations. Even among institutions, differences in terms of workload and working conditions may affect the perceived health status. It is important to note that nurses’ weekly working hours and night work are factors that affect both job satisfaction and life satisfaction, highlighting the need for improvement in these areas to increase satisfaction.

More than three-quarters of the participants in our study stated that they chose the nursing profession voluntarily. Approximately four-fifths of them found the nursing profession suitable for themselves. In a study by Duran et al., two-thirds of the participants chose the nursing profession voluntarily^23^. In a study by Mollaoğlu et al., more than three-fifths of the nurses chose their profession willingly^24^. A study conducted by Taycan et al. found that approximately three-fourths of the participants chose their profession voluntarily, and more than three-fourths found their profession suitable^25^. All these studies conducted in Türkiye, although conducted at different times and with different sample groups, show that those who chose the nursing profession mostly made this decision voluntarily and found their choice appropriate. These results may indicate a high level of interest and satisfaction in the nursing profession in Türkiye.

Those who chose the nursing profession willingly reached higher mean scores in the Index of Job Satisfaction and Life Satisfaction Scale than those who did not willingly choose the nursing profession. Similarly, those who found the nursing profession suitable for themselves had higher mean scores in both scales than those who did not find it suitable. In a study on healthcare workers, job satisfaction was higher in those who willingly chose their profession^26^. In a study conducted with midwives and nurses, the life satisfaction of those who willingly chose their profession was significantly higher than those who chose their profession involuntarily^27^. In a study conducted with newly graduated nurses, those who liked the nursing profession adapted to their jobs more quickly and had higher job satisfaction levels^28^. Those who prefer the nursing profession willingly and find it suitable for themselves are likely to be loyal to their long-term career goals, have a solution-oriented approach to professional difficulties, and have high motivation. For all these reasons, they obtain more life satisfaction and job satisfaction. When people freely choose their professions and make them suit themselves, they may like their work more, positively affecting job satisfaction. Satisfaction in work life can also affect the pleasure taken from life and lead to high levels of life satisfaction.

Approximately nine out of ten participants stated that their monthly income needed to be increased. Those who found their monthly income sufficient had higher scores on the Index of Job Satisfaction and Life Satisfaction Scale than those who found their monthly income insufficient. In a previous study involving nurses in another university hospital in Türkiye, one-third of the participants stated that their monthly earnings were insufficient^25^. In another study conducted in Türkiye, life satisfaction was positively correlated with income satisfaction^29^. According to a study conducted with public health nurses in Canada, salary is one of the most essential components of job satisfaction^30^. A study conducted with nurses in Spain found a positive relationship between economic crisis and burnout^31^. In a study conducted in Nigeria, salary was the most crucial determinant of nurses’ job satisfaction^32^. All these studies emphasize the importance of salary policies for employees. Salary is one of the most important factors that provide job satisfaction. In cases where monthly earnings are sufficient, people can meet their needs more efficiently. This situation has a positive effect on job satisfaction and life satisfaction. The fact that nurses are in financial distress should be handled carefully as it may affect the quality and sustainability of health services. Adequate monthly income significantly contributes to life satisfaction, allowing individuals to meet their needs more effectively and reducing financial stress. The difference between the two studies conducted in Türkiye in terms of income satisfaction may be attributed to the adverse economic conditions in the country over time.

Among the participants in our study, those aged 40 years and over were found to have higher job satisfaction scores than the participants aged under 40 years. Another study conducted with nurses in Türkiye found that the mean job satisfaction was lowest in the 19–24 age group and highest in the 40 and over age group^33^. A study conducted in Hong Kong found that age was positively associated with job satisfaction and mental comfort^34^. Birdi et al. also found a positive relationship between age and job satisfaction^35^. The parallelism between age and job satisfaction may have emerged due to experience, competence, professional and personal maturity, better adaptation to work, and more control.

Among our study participants, the mean score of the Index of Job Satisfaction of those who did not work at night was higher than those who worked at night. In a study conducted in China, the frequency of night shifts significantly affected job satisfaction^36^. According to a study conducted in Italy, nurses working night shifts had the lowest mean scores regarding job satisfaction^37^. Similarly, a study conducted in Hong Kong showed that nurses without night shifts had higher job satisfaction than nurses working at night^38^. Working at night affects the individual physically and psychologically as one of the causes of circadian rhythm disruption. Those who work at night may experience more stress and fatigue due to shift work organization. It may reduce job satisfaction. On the other hand, those who do not work at night may have higher job satisfaction because they have a better sleep pattern. For all these reasons, working at night negatively affects the mean scores of the Index of Job Satisfaction.

Among the nurses in our study, the mean scores of the Index of Job Satisfaction of those working outside the clinics were higher than those working there. In a study conducted by Klaus et al. using national data from the United States of America, job satisfaction in nurses was significantly related to the department in which they worked^39^. In a similar study conducted by Boyle et al., the groups with the highest job satisfaction were those working in rehabilitation and outpatient units, and the least satisfied groups were those working in emergency and surgical wards^40^. According to another study conducted in the United States of America, nurses’ job dissatisfaction was significantly related to the patient/nurse ratio^41^. The excessive workload and continuous long hours of nurses working in the wards may have led to these results. In addition, the fact that nurses working in wards experience processes requiring more intensive communication with patients and their relatives may have negatively affected job satisfaction.

The mean scores on the Job Satisfaction and Life Satisfaction Scale of the participants who rated their working conditions as very good/good were higher. A study conducted in Hong Kong observed that nurses’ general perceptions of their working environment were significantly related to job satisfaction^42^. A qualitative study on Saudi Arabian nurses reported that a stressful working environment was an important factor affecting job satisfaction^43^. In a study conducted in Jordan, a positive relationship was found between the working environment and the job satisfaction of nurses^44^. Employees with good working conditions generally enjoy their work more, are motivated, and have higher job satisfaction. On the other hand, poor working conditions may adversely affect job satisfaction. These may include job dissatisfaction, stress, burnout, health problems, and decreased performance.

Our study found a significant, positive, and moderate relationship between job and life satisfaction. Some basic ideas come to the fore in previous studies trying to explain the relationship between job and life satisfaction. The spillover model suggests that satisfaction or dissatisfaction in one area of an individual’s life will affect other areas, which is bidirectional^45^. A study conducted in Türkiye reported a positive relationship between job and life satisfaction^46^. A review examining the relationship between job and life satisfaction reported a positive relationship between job satisfaction and life satisfaction in more than 90% of the research group^47^. A positive relationship was observed between job satisfaction and happiness variables in Peruvian nurses^48^. These findings show that work and personal life affect each other, and this interaction is generally favorable. It is possible that employees spend a significant part of their lives at the workplace, and therefore, they may be affected by positive or negative situations related to work.

Our study has some strengths and limitations. We conducted this study with many participants, and the access percentage was also high. However, the fact that our study is a cross-sectional study conducted in a single center may have led to limitations in establishing causal links between the factors and generalizability. The questionnaire includes valid and reliable scales, but some of our data are based on the employees’ statements. Most participants were women and university graduates. This situation made it challenging to make comparisons about gender and education levels. However, this difficulty is common, observed in many studies, and related to the sociodemographic characteristics of the working nurse population.

## Conclusion

This study found a significant positive relationship between job and life satisfaction among nurses at Gazi University Faculty of Medicine Hospital. Job satisfaction was influenced by age, having children, working at night, working hours, work unit, and tenure. While smoking and chronic diseases affected life satisfaction, they did not influence job satisfaction. Key factors impacting job and life satisfaction included perceived health status, working hours, voluntary choice of profession and unit, and income adequacy.

Improving working conditions, reducing night shifts, and addressing salary issues will enhance job and life satisfaction among nurses. Ensuring nurses can choose their work unit and providing fair salaries will significantly contribute to their well-being. Policymakers should focus on strategies that address these factors to improve nurse retention and ensure high-quality healthcare services. Future research should explore specific areas of dissatisfaction to enhance working conditions and job satisfaction further.

## Data Availability

The datasets used and/or analysed during the current study available from the corresponding author upon reasonable request.
